# Determination and risk assessment of UV filters and benzotriazole UV stabilizers in wastewater from a wastewater treatment plant in Lüneburg, Germany

**DOI:** 10.1007/s10661-024-12853-2

**Published:** 2024-07-11

**Authors:** Akinranti S. Ajibola, Marco Reich, Klaus Kümmerer

**Affiliations:** 1 Institute of Sustainable Chemistry, Leuphana University Lüneburg, Universitätsallee 1, 21335 Lüneburg, Germany; 2 Analytical/Environmental Unit, Department of Chemistry, University of Ibadan, Ibadan, Nigeria

**Keywords:** UV absorbents, Emerging contaminants, Effluent, Influent, Personal care products, Ecotoxicological risk

## Abstract

UV filters and benzotriazole UV stabilizers are considered emerging contaminants in the environment. LC–MS/MS and GC–MS methods, involving a single solid phase extraction protocol, were developed and validated to determine eight UV filters and seven UV stabilizers, respectively in wastewater from a wastewater treatment plant (WWTP) in Lüneburg, Germany. The LC–MS/MS method exhibited extraction recoveries of ≥ 71% at six different fortification levels with limits of detection (LODs) range of 0.02 ng mL^−1^ – 0.09 ng mL^−1^. Extraction recoveries of 47 to 119% at six different fortification levels were obtained for the GC–MS method with LODs range of 0.01 – 0.09 ng mL^−1^. Among the UV filters, the highest mean concentration was determined for octocrylene (OCR) in influent (3.49 ng mL^−1^) while the highest mean concentration was measured for 2-hydroxy-4-octyloxybenzophenone (UV 531) in influent (0.44 ng mL^−1^) among the UV stabilizers. Potential risk to aquatic organisms was assessed by the risk quotient approach. Only OCR presented a high risk to aquatic invertebrates whereas 2-ethylhexyl 4-methoxycinnamate (EHMC) and 2-ethylhexyl salicylate (EHS) posed high risks to algae. Benzotriazole UV stabilizers presented negligible risks to aquatic invertebrates and fish. This work reports the detection of rarely studied 4-aminobenzoic acid (PABA) and UV 531 in WWTP influent and effluent. The occurrence and risk assessment of target benzotriazole UV stabilizers in wastewater from a German WWTP was demonstrated for the first time.

**Supplementary Information:**

The online version contains supplementary material available at 10.1007/s10661-024-12853-2.

## Introduction

In the past few years, the use of UV filters and benzotriazole UV stabilizers (collectively referred to as UV absorbents) in personal care products (PCPs) and technical applications has increased due to the growing concern about the link between sunlight exposure and skin cancer, as well as concerns about degradation of industrial products in the presence of ultraviolet radiation from sunlight (Gago-Ferrero et al., 2011; Montesdeoca-Esponda et al., 2013; Ruan et al., 2012). UV absorbents find applications in industrial materials and cosmetics worldwide to prevent photo-induced degradation in plastics and to protect human skin against the harmful effects of UV radiation (Apel et al., 2018a). Consequently, the extensive use of UV absorbents has already led to their frequent detection in many aquatic ecosystems (Cadena-Aizaga et al., 2020; Carve et al., 2021; Mao et al., 2019; Ramos et al., 2016).

UV filters and benzotriazole UV stabilizers have been listed as emerging contaminants in the environment (Montesdeoca-Esponda et al., 2019; Richardson, 2009). UV absorbents are an emerging risk due to their ubiquitous use in PCPs and technical applications such as additives in building materials, paints, automobile components, furniture, and polymeric materials (Carpinteiro et al., 2012; Ramos et al., 2016). UV filters and benzotriazole UV stabilizers have been found to pose potential threats to the ecological environment and human health such as endocrine-disrupting properties and acute toxicity (Fent et al., 2014; Rehfeld et al., 2018). This has led to the recognition of UV filters by the European Parliament as important organic contaminants in the aquatic environment (European Parliament, 2007; Molins-Degaldo et al., 2017a). Some benzotriazole UV stabilizers, especially UV 328, show specific target organ toxicity following repeated exposure (Brandt et al., 2016; ECHA, 2013). There are also indications that transformation products formed by the photolysis of some UV filters could be mutagenic and genotoxic (Westphal et al., 2020).

UV filters and benzotriazole UV stabilizers enter the environment through direct input from human activities, such as wash-off from skin and clothing during recreational activities, and indirect entry through wastewater effluents, industrial discharges, runoffs, and domestic uses (Cadena-Aizaga et al., 2020; Montesdeoca-Esponda et al., 2019;). Monitoring programs revealed that effluents from WWTPs are the main contributors to UV-filters and UV stabilizers contamination in the aquatic ecosystem (Ramos et al., 2016; Molins-Delgado et al., 2017b). Generally, there are limited studies on benzotriazole UV stabilizers in wastewater, probably because they tend to adsorb on sludge during wastewater treatment due to their relatively high Log K_ow_ values (Table S1, supplementary material). UV filters have been detected in German river water and sediments (Rodil & Moeder, 2008; Wick et al., 2010, 2016) and more recently in the sediment of European North and Baltic seas (Apel et al., 2018b). However, little is known about the determination and occurrence of these UV absorbents in wastewater from the German WWTPs. The few existing studies (Moeder et al., 2010; Rodil et al., 2009; Wick et al., 2010) on UV absorbents determination in wastewater from German WWTPs focused on only a few selected UV filters used in cosmetics. To our knowledge, no study exists to date on the determination of benzotriazole UV stabilizers in wastewater from German WWTPs. Hence, considering their wide usage, it is worthwhile to investigate the occurrence of UV filters and benzotriazole UV stabilizers in wastewater from the German WWTPs.

In this regard, developing sensitive analytical methods for accurately determining these compounds in wastewater matrices is necessary to assess their impact on the environment and their possible deleterious effects on human health. It is usually very challenging to extract simultaneously and efficiently micropollutants with widely differing partition coefficients (Log K_ow_) from wastewater. Wastewater is a complex environmental matrix and endogenous components can severely impair the accurate determination of micropollutants through matrix interferences. Thus, an efficient sample preparation coupled with a sensitive instrumental method is highly required for their accurate determination.

This study was, therefore, aimed at developing sensitive LC–MS/MS and GC–MS methods for the determination of UV filters and benzotriazole UV stabilizers, using a single solid phase extraction protocol that allows simultaneous extraction from wastewater fifteen multiclass UV filters and stabilizers with widely differing Log K_ow_ values (0.83 to 7.7) (Table S1, supplementary material). The compounds included in this work are UV filters allowed in cosmetics in the EU and UV stabilizers used in plastics, coatings, paints and textile applications (Brooke et al., 2008; Remberger et al., 2015). Moreover, some selected UV absorbents in this study are substances of interest within the large-scale ten-year cooperation project on biomonitoring between the German Federal Ministry for the Environment (BMUB) and the German Chemical Industry Association (VCI) (Kolossa-Gehring et al., 2017). The LC–MS/MS and GC–MS methods were validated to determine target UV filters and benzotriazole UV stabilizers. The methods were successfully applied to determine the concentration profiles of target UV filters and benzotriazole UV stabilizers in wastewater from a WWTP in Lüneburg, Germany. The first report on the occurrence and risk assessment of target benzotriazole UV stabilizers in wastewater from a German WWTP is provided.

## Materials and methods

### Chemicals and reagents

Analytical standards of 4-Aminobenzoic acid (PABA, 99%), 2-hydroxy- 4-methoxybenzophenone (BP3, 98%) and 2-hydroxy-4-octyloxybenzophenone (UV 531) were obtained from Alfa Aesar (Karlsruhe, Germany). Analytical standards of 2-ethylhexyl salicylate (EHS, 99%), Butylmethoxydibenzoylmethane or Avobenzone (AVO, ≥ 99%), 2-ethylhexyl 4-methoxycinnamate (EHMC, 98%), 3-(4-methylbenzylidene)camphor (4-MBC, 98%), octocrylene (OCR, 97%), octocrylene-d_15_ (OCR-d_15_), octyldimethylparaaminobenzoate (ODPABA, 98%), 2-(benzotriazol-2-yl)-4-methylphenol (UV P), 2-(2H-benzotriazol-2-yl)-4,6-bis(2-phenyl-2-propanyl)phenol (UV 234), 2-tert-butyl-6-(5-chlorobenzotriazol-2-yl)-4-methylphenol (UV 326), 2,4-ditert-butyl-6-(5-chlorobenzotriazol-2-yl)phenol (UV 327), 2-(benzotriazol-2-yl)-4,6-bis(2-methybutan-2-yl)phenol (UV 328) and 2-(benzotriazol-2-yl)-4(2,4,4-trimethylpentan-2yl)phenol (UV 329) were purchased from Sigma Aldrich (Steinheim, Germany). Allyl-benzotriazole was obtained from TCI Deutschland GmbH (Eschborn, Germany). The CAS number, structures, Log K_ow_ values, and chemical classes of the compounds are shown in Table S1 (Supplementary material). HPLC grade dichloromethane, ethyl acetate (> 99.5%) and cellulose filter paper ROTILABO® (type 113P, 150 mm) were purchased from Carl Roth GmbH (Karlsruhe, Germany), LC–MS grade methanol, acetonitrile, ammonium acetate, and formic acid were obtained from VWR (BDH Chemicals), GC–MS grade N-hexane was purchased from Merck KGaA (Darmstadt, Germany). Ultrapure water was produced using the Milli-Q water purification system.1 g/L stock solution of the individual compound and OCR-d_15_ was prepared in methanol. UV 234 and UV 326 were prepared in acetonitrile. Allyl-benzotriazole was prepared in acetonitrile/methanol (1:1). Working standard solution was prepared from the stock solution.

### Sampling and sample pretreatment

Wastewater samples were collected from the wastewater treatment plant in AGL Lüneburg, Germany (about 75,000 inhabitants). The wastewater treatment facility serves the northern city of Lüneburg and six additional nearby towns (Siemens worldwide references for the water industry, 2011). It receives up to 25,000 m^3^ of wastewater daily from residents, industrial and commercial customers (Siemens worldwide references for the water industry). About two-thirds of wastewater originates from private households, and one-third from industrial processes (Weinberg et al., 2011). It was designed to meet the needs of around 325,000 inhabitants (Siemens worldwide references for the water industry, 2011). The treatment plant is equipped with mechanical sewage treatment, followed by primary sedimentation basins, aeration tanks, secondary sedimentation basins, a biological phosphorus elimination system, and digestion towers (Weinberg et al., 2011). Effluent samples were collected after the biological treatment stage. Grab effluent samples were collected in December 2018 (first sampling), while grab influent and effluent samples were collected in January 2019 (second sampling). 25 L of wastewater (influent and effluent samples) were collected during each sampling. Wastewater samples (influent and effluent) were filtered using cellulose filter paper ROTILABO® (type 113P, 150 mm) and stored in the freezer at -20℃ before extraction.

### Solid phase extraction

In the optimized solid phase extraction protocol, Chromabond HLB cartridges were fitted on a vacuum apparatus. 500 mL filtered wastewater was spiked with internal standard OCR-d_15_ at 2 ng mL^−1^. SPE sorbent was conditioned with 5 mL ethylacetate/dichloromethane (1:1), 5 mL methanol followed by 5 mL ultrapure water. The wastewater sample was loaded on the SPE cartridge at a flow rate of 6 mL min^−1^. The sample bottle and SPE tubing were rinsed with 5 mL ultrapure water with applied pressure under a gentle vacuum to minimize adsorption. Cartridges were then washed with 5 mL ultrapure water and allowed to dry for 30 min under a vacuum. Elution was carried out with 2 × 10 mL ethylacetate/dichloromethane (1:1). The collected 20 mL of elution solvent was transferred into a centrifuge tube, vortex-mixed and divided into two equal volumes (approximately 10 mL for LC–MS/MS analysis and approximately 10 mL for GC–MS determination). The extract was evaporated to dryness under a gentle stream of nitrogen. The final extract was reconstituted with 0.5 mL acetonitrile/0.1% formic acid in water (1:1) for LC–MS/MS determinations and with 0.5 mL of n-hexane for GC–MS analyses.

### LC–MS/MS analysis

LC–MS/MS analysis was performed using an Agilent HPLC 1200 system (Agilent Technologies, Inc., Santa Clara, CA, USA) coupled to an Agilent 6430 triple quadrupole mass spectrometer (Agilent Technologies, Inc., Santa Clara, CA, USA). The mass spectrometer is equipped with an electrospray ionization source in both positive and negative modes. Chromatographic separation of target compounds was achieved on a Kinetex C18 column (100 × 2.1 mm, 2.6 µm). The column oven temperature was set at 35℃, and a sample injection volume of 5 µL was used. Mobile phases consisted of acetonitrile (A) and ultrapure water containing 0.1% formic acid (B) at a flow rate of 0.4 mL min^−1^. The gradient elution was set as follows: at 0 min 50% A; at 6 min 50% A; at 6.1 min 80% A; at 8 min 100% A; at 12 min 100% A; at 12.1 min 50% A; and at 17 min 50% A.

Mass spectrometric analysis of target compounds was carried out with a triple quadrupole mass spectrometer using an electrospray ionization source in positive mode (ESI^+^). In order to obtain MS/MS transitions with a suitable sensitivity for identification and quantification of the target UV absorbents, MS/MS parameters (collision energy, fragmentor voltage, and capillary voltage) were optimized for each target compound using the Agilent Optimizer software. 1 mg L^−1^ standard solution of each compound was prepared in acetonitrile/water (1:1). Direct injection (without column) of 1 mg L^−1^ standard solution was used to optimize at a flow rate of 0.4 mL min^−1^ and injection volume of 5 µL. Different compositions of mobile phase solvents were tested: ACN/0.1% formic acid in water (50:50) and ACN/1 mM ammonium acetate in water (50:50). Optimization was carried out with each mobile phase using the electrospray ionization (ESI) in both positive and negative modes.

The capillary voltage was 4000 V, and the nebulizer pressure was 50 psi. The drying gas flow and drying gas temperature were 8 L min^−1^ and 325℃, respectively. Data acquisition was performed in multiple reaction monitoring (MRM) mode and processed using the MassHunter software version B.08.02 by Agilent Technologies Inc. Two MRM transitions were monitored for each compound. The ion transition with the highest abundance was selected for quantification (quantifier), while the ion transition with the second highest abundance was selected for confirmation (qualifier). The fragmentation voltage, collision energy, and multiple reaction monitoring mode transitions were optimized for each compound. The optimized values for the parameters are presented in Table 1. Internal standard calibration was applied to quantify target analytes.

**Table 1:** Retention time and optimized MS/MS Parameters of target UV filters for LC-MS/MS^a^

Compound	Retention time (min)	Precursor ion (*m/z*)	Product ion (*m/z*)	Collision energy (*v*)	Fragmentation Voltage (*v*)
**AVO**	1.69	**311.2**	**135.1**	20	138
		311.2	161.1	24	
**BP3**	1.52	**229.1**	**151.1**	20	138
		229.1	77.1	52	
**EHMC**	4.34	**291.2**	**161.1**	20	59
		291.2	179.1	8	
**EHS**	5.14	**251.2**	**139.2**	8	64
		251.2	121	20	
**4-MBC**	2.54	**255.2**	**105.1**	32	98
		255.2	77.1	76	
**OCR**	4.31	**362.2**	**250.1**	8	69
		362.2	232.1	20	
**OCR-d**_**15**_	4.18	**377.3**	**232.1**	20	128
		377.3	206.8	16	
**ODPABA**	4.29	**278.2**	**166.1**	20	143
		278.2	151.1	36	
**PABA**	0.99	**138.1**	**65.1**	28	108
		138.1	77.1	24	

^a^Transitions in bold were used for quantification

### GC–MS analysis

**Fig. 1:**
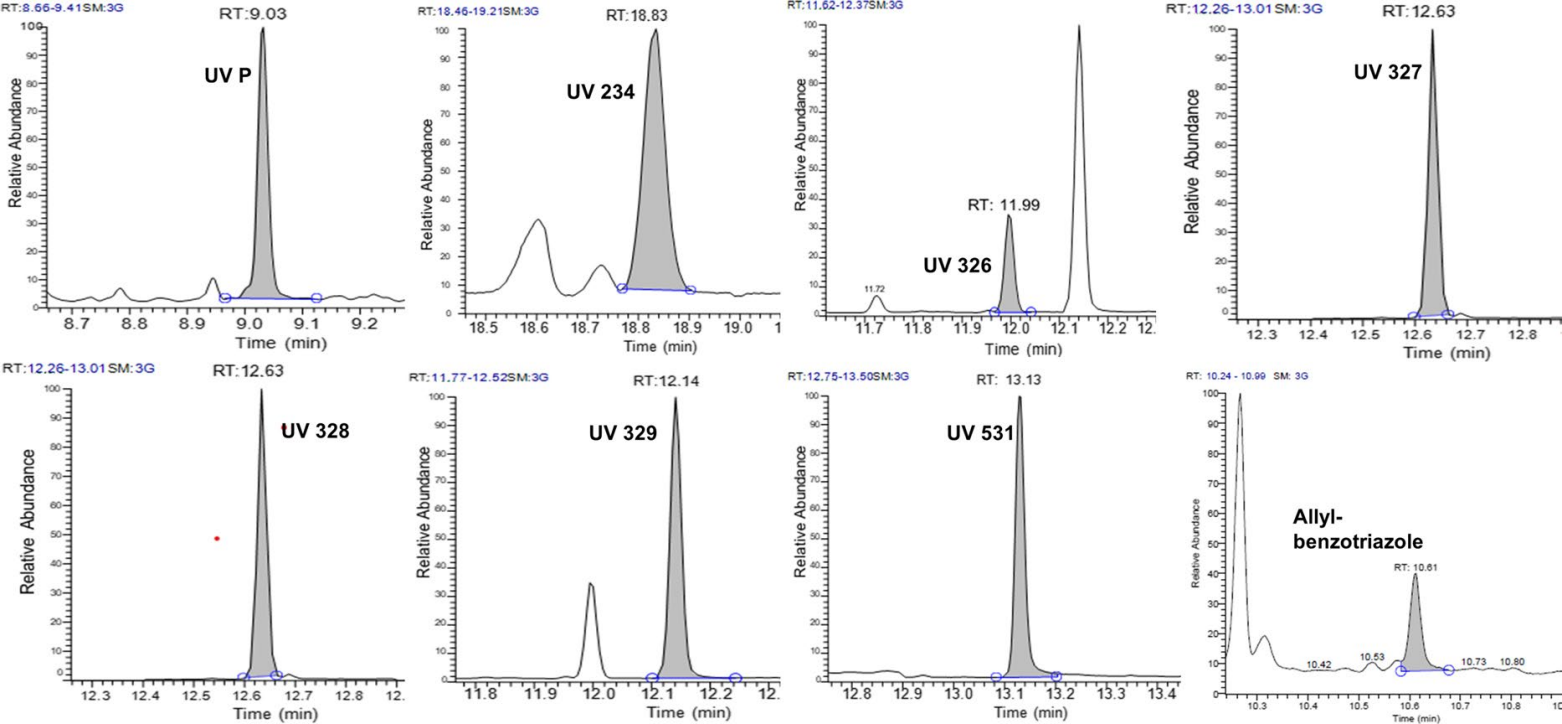
GC–MS chromatograms of target benzotriazole UV stabilizers (500 ng mL^−1^) and allyl-benzotriazole (50 ng mL^−1^) in wastewater extract spiked after extraction

GC–MS analyses were performed on a TRACE 1310 Series gas chromatograph (Thermo Scientific™) equipped with a TriPlus RSH autosampler and coupled to a ISQ Series (Thermo) single quadrupole mass spectrometer operating in electron impact ionization mode (EI, 70 eV). Separation of analytes was achieved on OPTIMA®-5MS capillary column of 30 m × 0.25 mm inner diameter and 0.25 μm film thickness (Macherey–Nagel, Germany). Helium was used as a carrier gas at a flow rate of 1 mL min^−1^. A split/splitless injector was used in a splitless mode with a splitless time of 1.0 min. The injector temperature was 300 ℃ while the injection volume was 1 µL. The oven temperature program was as follows: initial temperature of 80 ℃ held for 1 min, ramped at 25℃/min to 230 ℃ and held for 1 min, ramped at 15℃/min to 260 ℃ and held for 1 min, ramped at 20℃/min to 310 ℃ and held for 8 min. The ion source and MS transfer line temperatures were 280 ℃ and 300 ℃, respectively. MS Full scan mode was initially used to identify the retention time and to select the fragment ions of each compound. 1 mg L^−1^ standard mixture of all compounds was injected. Mass spectral library (NIST) was used for confirmation of each compound’s ions included in the database. A SIM mode, with improved sensitivity, was used for quantification based on the retention times and three fragment ions with the highest intensities for each compound. The most intense ion was used for quantification. The retention time and monitored SIM ions of target compounds are presented in Table 2. Matrix-matched calibration was applied for validation and quantification of the analytes. GC–MS chromatograms are shown in Fig. 1.

**Table 2:** Retention time and monitored SIM ions of target UV stabilizers for GC-MS^a^

Compound	Retention time (min)	SIM ions (m/z)
UV P	9.02	**225.09**, 168.08, 196.08
UV 234	18.84	**432.19**, 447.2, 342.1
UV 326	11.99	**300.08**, 315.09, 272.09
UV 327	12.63	**342.13**, 357.15, 344.14
UV 328	12.58	**322.19**, 351.22, 252.13
UV 329	12.13	**252.10**, 323.19, 253.13
UV 531	13.13	**213.07**, 326.19, 214.09
Allyl-benzotriazole	10.60	**250.09**, 265.12, 145.06

^a^ions in bold were used for quantification

### Quality assurance/control (QA/QC)

Procedural blanks and instrumental blanks were used to monitor any contamination during the analytical procedure and for instrumental performance. Internal standard calibration using OCR-d_15_ was used for quantification in LC–MS/MS analysis while matrix-matched calibration was used for quantification in GC–MS determinations. The personnel always wore gloves during the sample preparation and did not use any personal care products containing the target UV absorbents. To minimize the adsorption of target compounds, the sample bottle and SPE tubing were rinsed with 5 mL ultrapure water with applied pressure under a gentle vacuum.

### Method validation

The LC–MS/MS and GC–MS methods were validated for analytical quality parameters: extraction recovery, overall recovery, linearity, precision, limits of detection (LODs), limits of quantification (LOQs), and matrix effect. Recoveries were determined at different fortification levels ranging from low spiking concentration (0.02 ng mL^−1^) to high spiking concentration (2.0 ng mL^−1^). Expected concentration levels in the final extracts ranged from 10 ng mL^−1^ (initial spike at 0.02 ng mL^−1^) to 1000 ng mL^−1^ (initial spike at 2.0 ng mL^−1^). Extraction recoveries were determined by comparing the responses of pre-extraction spiked samples with the responses of the corresponding post-extraction spiked samples. Overall recoveries were calculated by comparing the responses of pre-extraction spiked samples to the responses of the corresponding standard solution in pure solvent. The matrix effect was evaluated by comparing the responses of post-extraction spiked samples with the responses obtained for the corresponding standard solution in pure solvent. The numbers of sample replicates for assessing the recoveries, precision and matrix effect were between 4 and 6, depending on fortification level.

Linearity was assessed by plotting a calibration curve for each compound using the internal standard calibration (OCR-d_15_) for LC–MS/MS determinations and matrix matched calibration for GC–MS analysis. The calibration curves were constructed at concentration levels from 10 ng mL^−1^ to 1000 ng mL^−1^ covering six different calibration points. Precision was appraised by carrying out replicate determinations of target compounds in pre-extraction spiked samples at different spiking concentration levels. Precision was expressed as the relative standard deviation (% RSD) of the results of replicate determinations for each fortification level. Method LODs and LOQs for LC–MS/MS and GC–MS analyses were determined according to the procedure by the United States Environmental Protection Agency (USEPA 2017). This was carried out by analysing replicate effluent samples spiked with the lowest concentration level at which the compound can be reliably detected (0.02 ng mL^−1^ before extraction corresponding to 10 ng mL^−1^ in final extract or 0.1 ng mL^−1^ before extraction corresponding to 50 ng mL^−1^ in final extract). The method LOD was then computed as follows in Eq. (1):

1 $$\text{LOD}={\text{t}}_{\left(\text{n}-\text{1,1}-a=0.99\right)}{\text{S}}_{\text{s}}$$

where t_(n-1, 1-α=0.99)_ is the Student’s *t*-value appropriate for a single-tailed 99th percentile *t* statistic and a standard deviation estimate with n-1 degrees of freedom; S_s_ is the sample standard deviation of the replicate spiked sample analyses.

The method LOQ was calculated as follows in Eq. (2):

2 $$\text{LOQ}=10\text{ x }{\text{S}}_{\text{s}}$$

where S_s_ is the sample standard deviation of the replicate spiked sample analyses.

### Environmental risk assessment

The potential risk of the presence of the investigated UV filters and benzotriazole UV stabilizers to aquatic organisms and ecosystems was assessed using the risk quotient (RQ) approach (Allinson et al., 2018). The risk quotient was evaluated by dividing the measured environmental concentration (MEC) of each UV absorbent in effluent samples by the predicted–no-effect- concentration (PNEC). The maximum concentration measured for each compound in the effluent was used as MEC to determine the worst-case scenario. In cases where the concentrations of UV absorbents in effluent samples were below the LOQ, the value of LOQ was used as MEC**.** No**-**effect-concentration (NOEC) data on the chronic (long-term) ecotoxicological effects for three trophic levels (fish, aquatic invertebrates, and algae) were obtained from the literature (Allinson et al., 2018) and are presented in Table S2 (Supplementary material). Short-term EC_50_ (lethal effect) data were used where NOEC data was not available (Allinson et al., 2018). The PNEC values were calculated by dividing the NOEC or EC_50_ value by an assessment factor. An assessment factor of 50 was used for only BP3, for which chronic data was available from three trophic levels, but the effect level used was an acute value, while an assessment factor of 100 was used for all other compounds (Table S2, Supplementary material). RQ of < 0.01 implies negligible risk; RQ ≥ 0.01 indicates low risk; RQ ≥ 0.1 shows moderate risk, while RQ ≥ 1 indicates high risk (Khare et al., 2023).

## Results and discussion

### Optimization of solid phase extraction protocol

A recovery test of the target compounds was initially carried out using ultrapure water. Two different SPE cartridges were tested for extraction efficiency: Strata X-CW (500 mg, 6 mL) from Phenomenex and Chromabond HLB (200 mg, 3 mL) from Macherey–Nagel. Strata X-CW is a polymeric weak cation exchange sorbent, while Chromabond HLB is a hydrophilic-lipophilic balanced N-vinylpyrrolidone-divinylbenzene copolymer. A preliminary SPE procedure during optimization was developed using ultrapure water, and it is presented in the supplementary material. The results of the extraction efficiencies in these preliminary experiments were calculated without internal standards (absolute recoveries). The peak area ratio was used for calculating recovery. Based on the results from the preliminary experiments, both Chromabond HLB and Strata X-CW cartridges provided similar absolute extraction recoveries for most of the target compounds in both LC–MS/MS (Table S3, Supplementary material) and GC–MS determinations (Table S4, Supplementary material). Except for BP3, 4-MBC, and UV P, which had close to 100% recoveries using the Chromabond HLB cartridges, extraction recoveries ranged from 29% (OCR) to 70% (ODPABA) for the compounds analyzed by LC–MS/MS. Absolute extraction recoveries of 29% (OCR) – 81% (PABA) were obtained for Strata X-CW cartridges in LC–MS/MS determinations.

For the UV stabilizers analyzed by GC–MS, low extraction recoveries of less than 24% from ultrapure water were generally obtained for the most hydrophobic of the target compounds using either the Chromabond HLB cartridges or Strata X-CW cartridges (Table S3, Supplementary material). It should be noted that the spiking concentration level of ultrapure water during the optimization was 1000 ng mL^−1^ (in the final extract) (Table S3, supplementary material), which is considered a high fortification level in this study. Carpinteiro et al. (2010) also obtained only 5% recovery in ultrapure water for hydrophobic UV stabilizers UV 327 and UV 328 using solid phase extraction. In GC–MS determinations, better extraction recoveries from ultrapure water were obtained for target compounds having lower Log K_ow_ values (less hydrophobic) than the most hydrophobic, with up to 94% (Chromabond HLB) and 87% (Strata X-CW) for UV P (the most polar benzotriazole UV stabilizer). However, substantial matrix ion enhancement was evident with Strata X-CW cartridges for the compounds analyzed by GC–MS (Table S4, supplementary material)). In contrast, small matrix ion suppression (for ultrapure water) of most target compounds was observed in LC–MS/MS determinations using both Chromabond HLB and Strata X-CW SPE cartridges (Table S3, supplementary material). Therefore, subsequent optimization with wastewater for recovery improvement was carried out using Chromabond HLB cartridges.

**Fig. 2:**
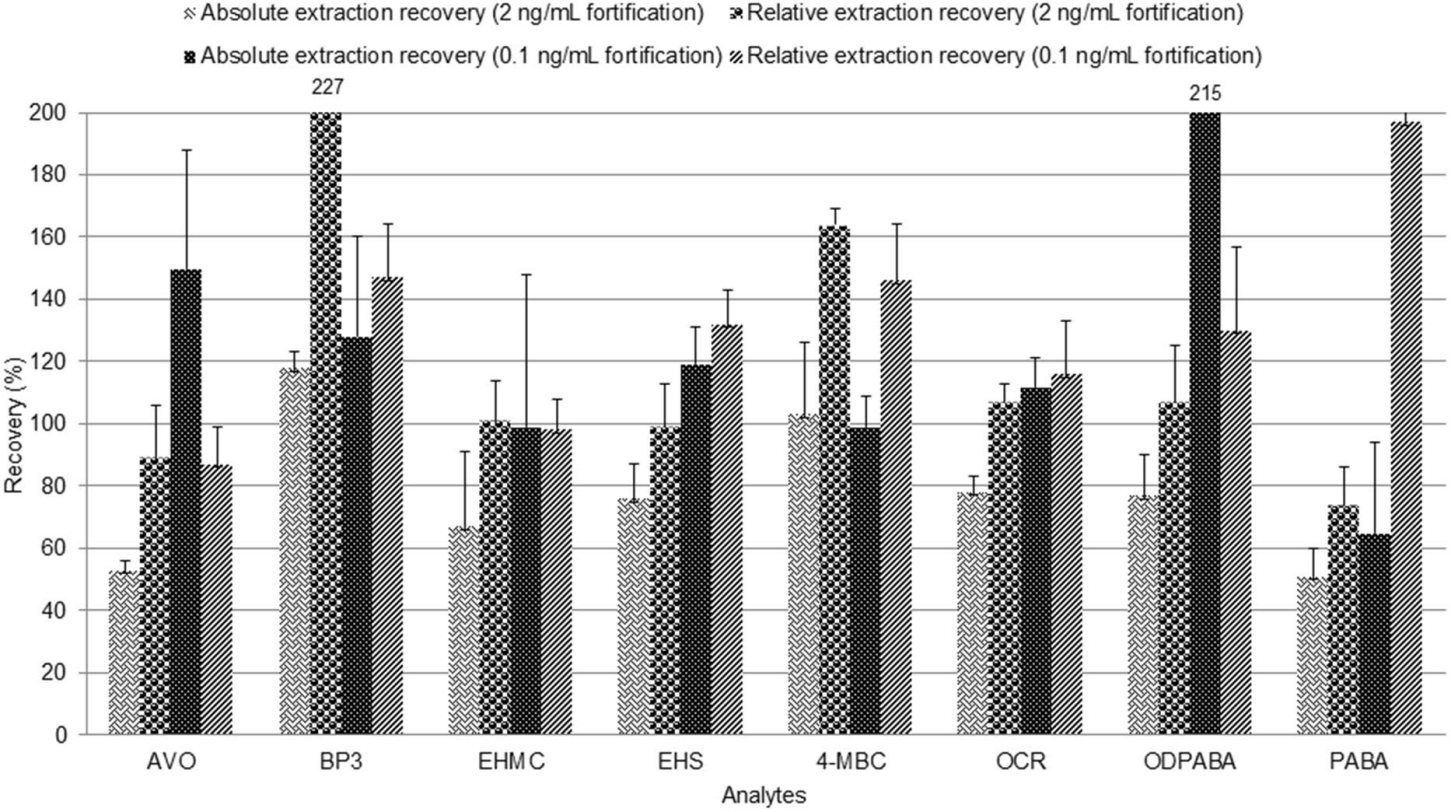
Comparison of absolute extraction recoveries and relative extraction recoveries in wastewater at different fortification levels (LC–MS/MS method)

SPE optimization with wastewater was carried out at two fortification levels of 0.1 ng mL^−1^ and 2.0 ng mL^−1^. The optimized SPE procedure using wastewater is described above in the section on ‘Solid phase extraction.’ For the target UV filters analyzed by LC–MS/MS, absolute extraction recoveries at 2.0 ng mL^−1^ spiking level ranged from 51% (PABA) to 118% (BP3), while at 0.1 ng mL^−1^ fortification absolute extraction recoveries ranged from 65% (PABA) to 215% (ODPABA) as shown in Fig. 2. An improvement in the extraction efficiencies was observed in comparison with the preliminary SPE procedure using ultrapure water, especially for OCR. In the case of UV stabilizers analyzed by GC–MS, absolute extraction recoveries were also improved for most compounds, especially for the most hydrophobic compounds, which had very low recoveries in the preliminary SPE procedure using ultrapure water. Absolute extraction recoveries of UV stabilizers at 2.0 ng mL^−1^ spiking level ranged from 42% (UV 327 & UV 328) to 87% (UV P) whereas the absolute extraction recoveries at 0.1 ng mL^−1^ fortification ranged from 11% (UV 328) to 49% (UV 326). A larger volume of eluting solvent (20 mL ethylacetate/dichloromethane (1:1)) used in the procedure for the optimization with wastewater in contrast to 10 mL eluting solvent used for the optimization with ultrapure water may be responsible for the higher recoveries in wastewater than in ultrapure water, since more analytes adsorbed on the surface of the sorbent would be desorbed and eluted.

Absolute extraction recoveries (without internal standard) were compared with relative extraction recoveries (with internal standard) for the LC–MS/MS analysis using OCR-d_15_ as the internal standard. Figure 2 shows the results of absolute and relative recoveries with LC–MS/MS at 0.1 ng mL^−1^ and 2.0 ng mL^−1^ spiking concentrations. Using OCR-d_15_ as an internal standard compensated for any loss of the compounds during the sample preparation step, leading to an improvement in the extraction recoveries for most UV filters, including OCR, AVO, PABA, EHMC and EHS. The applicability of OCR-d_15_ for matrix effect reduction and the influence of matrix effect on the overall recovery in LC–MS/MS determinations are discussed later under the sub-section*** ‘***Matrix effect in LC–MS/MS.’

#### LC–MS/MS

Separation of the compounds was carried out by testing two different chromatographic columns: Nucleoshell RP 18 Plus (250 × 3 mm, 2.7 μm) from Macherey–Nagel, GmbH and Kinetex C18 (100 × 2.1 mm, 2.6 μm) from Phenomenex. Varying compositions of the mobile phase solvents in the isocratic elution mode were tested. The following mobile phase solvents compositions were experimented in the isocratic elution mode: Methanol/0.1% formic acid in water (70:30), acetonitrile/0.1% formic acid in water (70:30), acetonitrile/0.1% formic acid in water (30:70), acetonitrile/0.1% formic acid in water (50:50) and 0.01% formic acid in acetonitrile/0.1% formic acid in water (50:50). Under the experimented conditions, Kinetex C18 column provided slightly higher peak areas than the Nucleoshell RP 18 Plus column for most of the UV filters. Based on these preliminary experiments Kinetex column was selected. Using the Kinetex column, acetonitrile/0.1% formic acid in water (50:50) in the isocratic elution mode and at a flow rate of 0.4 mL min^−1^ provided resolved peaks within 10 min of elution time. However, some benzotriazole UV stabilizers were not eluted even when more extended elution program of twenty minutes was used. It should be noted that these non-eluted benzotriazole UV stabilizers under these chromatographic conditions are hydrophobic (Log Kow ≥ 5.6) (Table S1, supplementary material). In order to prevent possible carryover of analytes and column contamination due to matrix components between two successive injections, a gradient elution program was afterward designed. The gradient elution program allowed the organic content (acetonitrile) to be raised to 100% without affecting the resolution of the chromatographic peaks and sensitivity in the mass spectrometer. The gradient elution program is already given in the section ***‘***LC–MS/MS Analysis’ above. With the gradient elution program, all target analytes were eluted within 6 min, and a total run time of 17 min, including re-equilibration, was achieved.

### Optimization of MS/MS parameters

**Fig. 3:**
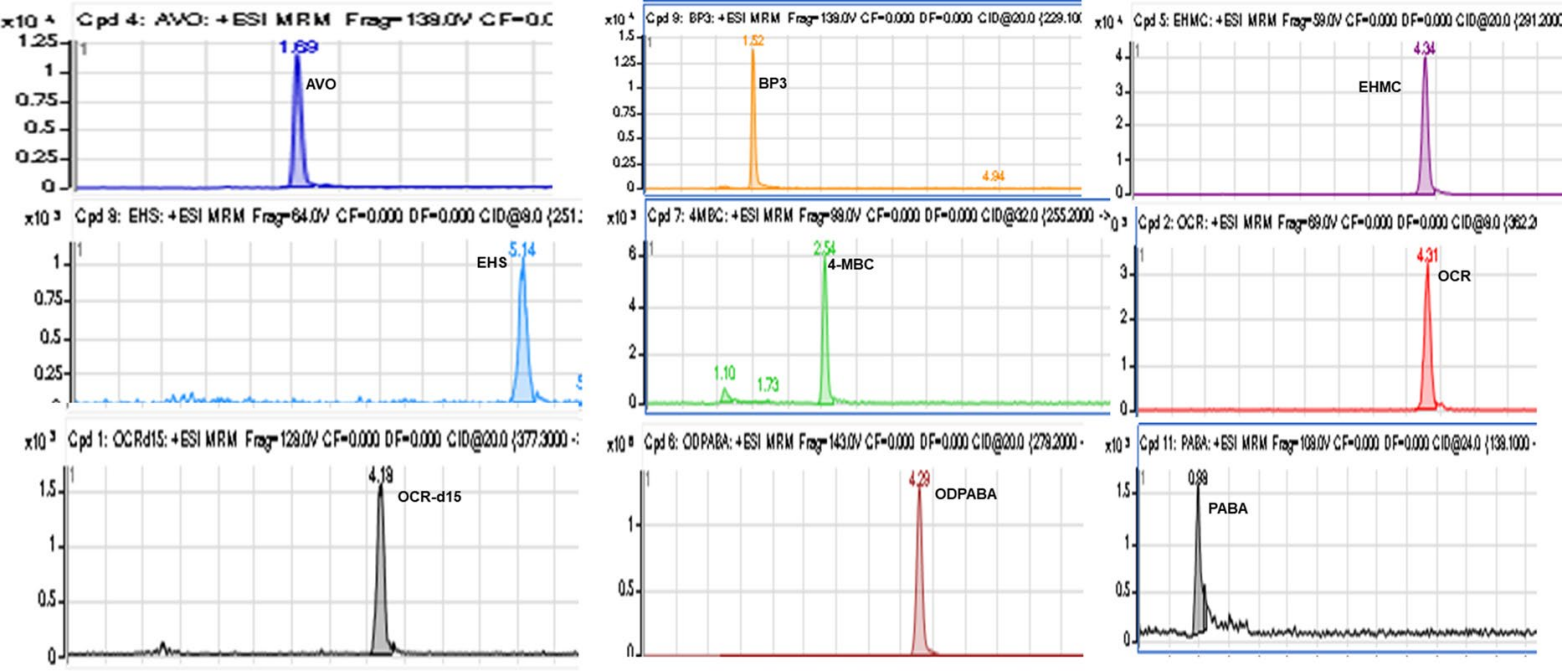
MRM (LC–MS/MS) chromatograms of target UV filters in wastewater extract spiked at 500 ng mL^−1^ after extraction

Ion intensities were generally lower in the negative mode than in the positive mode, using either ACN/0.1% formic acid or ACN/1 mM ammonium acetate as eluent. Acetonitrile/0.1% formic acid in water (50:50) in ESI-positive mode produced the highest intensities for most of the target UV absorbents. However, only a slight improvement was observed for some compounds, especially the most hydrophobic benzotriazole UV stabilizers. Therefore, GC–MS instrumentation was proposed for the analysis of these hydrophobic UV-stabilizers. The MS/MS chromatograms of the UV filters analyzed by LC–MS/MS are shown in Fig. 3.

## Analytical performance parameters

### LC–MS/MS method

The analytical quality parameters of the LC–MS/MS method are presented in Table 3. The LC–MS/MS method exhibited good linearity with coefficients of determination (r^2^) higher than 0.98 for all compounds. LOD values of the LC–MS/MS method ranged from 0.02 ng mL^−1^ to 0.09 ng mL^−1^ while LOQ values ranged from 0.06 ng mL^−1^ to 0.31 ng mL^−1^. The results of extraction recovery and overall recovery at six different fortification levels (ranging from low to high concentration levels) are shown in Table 3. At a low fortification level of 0.02 ng mL^−1^ extraction recoveries ranged from 96% (AVO) to 136% (PABA), whereas extraction recoveries at a high fortification level of 2.0 ng mL^−1^ ranged from 74% (PABA) to 227% (BP3). The overall recoveries of the method at a low fortification level of 0.02 ng mL^−1^ ranged from 95% (AVO) to 170% (OCR), while the overall recoveries at a high fortification level of 2.0 ng mL^−1^ ranged from 6% (PABA) to 126% (BP3 and 4-MBC). The relatively lower overall recoveries for PABA and AVO at high fortification levels could be attributed to ion suppressions in the mass spectrometer. This is discussed further under the section ‘Matrix effect in LC–MS/MS’. The LC–MS/MS method exhibited good precision with %RSD values of 3% to 22%, except for 4-MBC at 0.2 ng mL^−1^ fortification level only, in which a %RSD value of 29% was obtained.

**Table 3:** Analytical performance parameters of LC–MS/MS method for target UV absorbents

Compound	Extraction Recovery (%) (mean ± SD)						Overall Recovery (%) (mean ± SD)						Precision						LOD (ng mL^-1^)	LOQ (ng mL^-1^)	r^2^
													RSD (%), *n*= 4	RSD (%), *n*= 6	RSD (%), *n*= 4	RSD (%), *n*= 6	RSD (%), *n*= 5	RSD (%), *n*= 4			
	0.02 ng mL^-1^	0.1 ng mL^-1^	0.2 ng mL^-1^	0.5 ng mL^-1^	1.0 ng mL^-1^	2.0 ng mL^-1^	0.02 ng mL^-1^	0.1 ng mL^-1^	0.2 ng mL^-1^	0.5 ng mL^-1^	1.0 ng mL^-1^	2.0 ng mL^-1^	0.02 ng mL^-1^	0.1 ng mL^-1^	0.2 ng mL^-1^	0.5 ng mL^-1^	1.0 ng mL^-1^	2.0 ng mL^-1^			
**AVO**	96 ± 4	87 ± 12	79 ± 11	71 ± 10	85 ± 5	89 ± 17	95 ± 4	70 ± 9	60 ± 8	41 ± 2	45 ± 10	38 ± 7	4	13	14	14	6	19	0.02	0.06	0.9884
**BP3**	122 ± 8	147 ± 17	173 ± 10	176 ± 19	249 ± 38	227 ± 27	128 ± 9	133 ± 15	141 ± 8	185 ± 20	143 ± 22	126 ± 15	7	11	6	11	15	12	0.03	0.09	0.9850
**EHMC**	97 ± 3	98 ± 10	108 ± 9	86 ± 3	104 ± 11	101 ± 13	98 ± 3	93 ± 9	105 ± 9	90 ± 10	93 ± 13	80 ± 11	3	10	9	4	11	13	0.05	0.16	0.9921
**EHS**	108 ± 24	132 ± 11	102 ± 20	98 ± 13	102 ± 6	99 ± 14	114 ± 25	127 ± 10	108 ± 21	110 ± 15	76 ± 5	62 ± 9	22	8	19	13	6	14	0.09	0.31	0.9813
**4-MBC**	105 ± 11	146 ± 18	151 ± 44	156 ± 16	187 ± 22	164 ± 5	98 ± 11	120 ± 15	136 ± 40	181 ± 19	147 ± 17	126 ± 4	11	10	29	11	12	3	0.03	0.11	0.9834
**OCR**	91 ± 11	116 ± 17	122 ± 11	103 ± 7	130 ± 21	107 ± 6	170 ± 20	129 ± 19	127 ± 12	112 ± 8	96 ± 16	75 ± 4	12	15	9	7	17	5	0.08	0.28	0.9933
**ODPABA**	118 ± 17	130 ± 27	135 ± 16	101 ± 7	116 ± 24	107 ± 18	146 ± 21	137 ± 28	156 ± 18	149 ± 10	114 ± 23	104 ± 17	15	20	12	6	21	17	0.03	0.11	0.9963
**PABA**	136 ± 15	197 ± 36	109 ± 3	92 ± 10	103 ± 5	74 ± 12	130 ± 14	126 ± 23	45 ± 1	24 ± 2	11 ± 0.5	6 ± 1	11	18	3	10	4	16	0.03	0.10	0.9917

### Matrix effect in LC–MS/MS

**Fig. 4:**
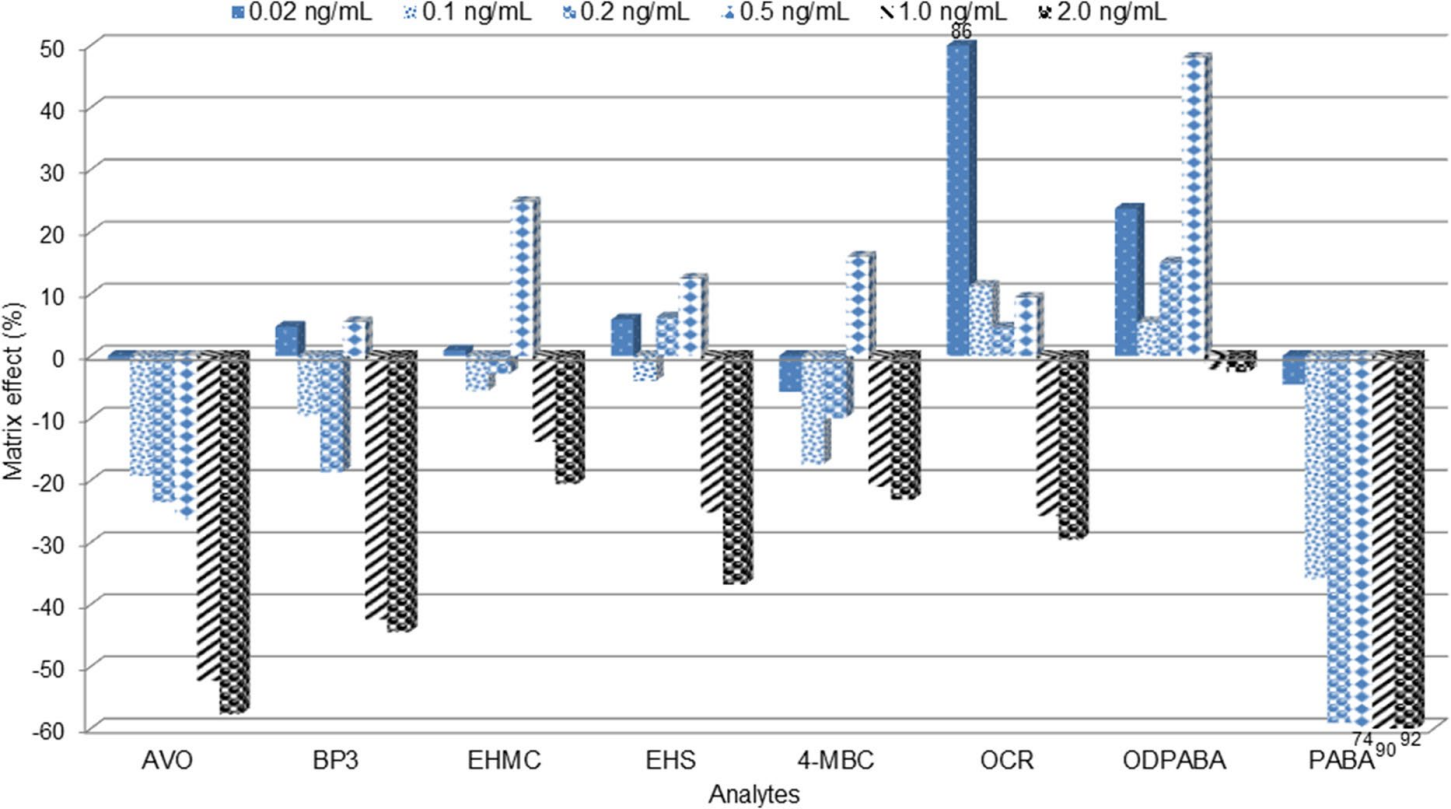
Matrix effects in LC–MS/MS method at different fortification levels

One major drawback of electrospray ionization (ESI) is its susceptibility to enhancing or suppressing analyte ions due to the matrix effect. The matrix effect was evaluated at the six different fortification levels (spanning the low, medium and high concentrations) in LC–MS/MS and the result is shown in Fig. 4. At low fortification levels no significant matrix effect was evident for all compounds (< 25%) except OCR, which exhibited substantial ion enhancement (86%) at 0.02 ng mL^−1^. Significant matrix ion suppression was evident for PABA, AVO and BP3 at high fortification levels of 1.0 ng mL^−1^ and 2.0 ng mL^−1^. This phenomenon of matrix ion suppression may be responsible for the relatively lower overall recoveries of PABA and AVO at these high fortification levels, as earlier mentioned. The applicability of OCR-d_15_ as an internal standard for matrix effect reduction in LC–MS/MS was demonstrated, as shown in Fig. S1 (supplementary material). The results showed that using OCR-d_15_ as an internal standard effectively reduced the matrix effects for all target UV filters except PABA and AVO, which exhibited only a small reduction in matrix ion suppression at medium and high fortification levels.

### GC–MS method

The analytical quality parameters of the GC–MS method are presented in Table 4. Good linearity was achieved for all UV stabilizers analyzed by GC–MS with coefficients of determination (r^2^) higher than 0.99. The GC–MS method’s LOD ranged from 0.01 ng mL^−1^ (for UV 328) to 0.09 ng mL^−1^ (for UV 234), while method LOQ ranged from 0.04 ng mL^−1^ (for UV 328) to 0.28 ng mL^−1^ (for UV 234). The achieved LODs and LOQs for the GC–MS method were in the same order or lower than previously reported studies (Kameda et al., 2011; Zhao et al., 2017; Carpinteiro et al., 2012; Wang et al., 2018; Tang et al., 2018; Montesdeoca-Esponda et al., 2012; Montesdeoca-Esponda et al., 2013, Liu et al., 2014; Castilloux et al., 2022; Kotowska et al., 2021; Zhang et al., 2021; Khare et al., 2023; Zhao et al., 2023). Precision at the six different fortification levels (covering low, medium and high concentrations) ranged from 4 to 33% (%RSD) in the GC–MS method. Extraction recoveries of benzotriazole UV stabilizers at low spiking concentrations 0.02 ng mL^−1^ and 0.1 ng mL^−1^ ranged from 47 to 115% while the extraction recoveries at medium fortification levels 0.2 ng mL^−1^, 0.5 ng mL^−1^ and high fortification levels 1.0 ng mL^−1^ and 2.0 ng mL^−1^ ranged from 66 to 102% and 94% to 119%, respectively. Moreover, the overall recoveries at low fortification levels 0.02 ng mL^−1^ and 0.1 ng mL^−1^ ranged from 40 to 132% whereas the overall recoveries at medium fortification levels 0.2 ng mL^−1^ and 0.5 ng mL^−1^ and high fortification levels 1.0 ng mL^−1^ and 2.0 ng mL^−1^ ranged from 64 to 105% and 82% to 103%, respectively. Generally, good extraction and overall recoveries were obtained except at the low fortification levels 0.02 ng mL^−1^ and 0.1 ng mL^−1^ for which extraction recoveries around 50% were obtained for UV 234, UV 326, UV 327 and UV 329. At low concentrations, the recovery of an analyte may not be independent of its concentration because a proportion of the analyte may be unrecoverable by virtue of irreversible adsorption on surfaces (Thompson et al., 1999). However, once the adsorption sites are all occupied, which could occur at a particular concentration of analyte, no further loss is likely at higher concentrations (Thompson et al., 1999). This could be the reason why good recoveries were generally obtained at higher fortification levels for the benzotriazole UV stabilizers.

**Table 4:** Analytical performance parameters of GC–MS method for target UV absorbent

Compound	Extraction Recovery (%) (mean ± SD)						Overall Recovery (%) (mean ± SD)						Precision						LOD (ng mL^-1^)	LOQ (ng mL^-1^)	r^2^
													RSD (%), *n*= 4	RSD %), *n*= 6	RSD (%), *n*= 4	RSD (%), *n*= 4	RSD (%), *n*= 6	RSD (%), *n*= 4			
	0.02 ng mL^-1^	0.1 ng mL^-1^	0.2 ng mL^-1^	0.5 ng mL^-1^	1.0 ng mL^-1^	2.0 ng mL^-1^	0.02 ng mL^-1^	0.1 ng mL^-1^	0.2 ng mL^-1^	0.5 ng mL^-1^	1.0 ng mL^-1^	2.0 ng mL^-1^	0.02 ng mL^-1^	0.1 ng mL^-1^	0.2 ng mL^-1^	0.5 ng mL^-1^	1.0 ng mL^-1^	2.0 ng mL^-1^			
**UV P**	64 ± 21	86 ± 18	88 ± 15	100 ± 10	119 ± 13	101 ± 13	81 ± 27	65 ± 14	85 ± 15	105 ± 11	98 ± 11	100 ± 13	33	21	17	10	11	13	0.03	0.10	0.9976
**UV 234**	54 ± 17	53 ± 12	66 ± 13	92 ± 15	95 ± 17	96 ± 11	82 ± 26	51 ± 11	64 ± 13	82 ± 14	87 ± 16	99 ± 11	31	22	20	17	18	11	0.09	0.28	0.9980
**UV 326**	55 ± 17	47 ± 11	102 ± 20	96 ± 15	96 ± 13	100 ± 7	94 ± 30	40 ± 10	88 ± 18	91 ± 15	93 ± 12	101 ± 7	32	24	20	16	13	7	0.04	0.13	0.9935
**UV 327**	57 ± 3	65 ± 11	89 ± 25	94 ± 13	97 ± 8	99 ± 7	84 ± 4	54 ± 9	74 ± 21	91 ± 12	94 ± 8	100 ± 8	4	33	24	12	7	6	0.04	0.12	0.9925
**UV 328**	91 ± 7	71 ± 12	87 ± 17	91 ± 10	94 ± 9	100 ± 5	133 ± 11	61 ± 3	93 ± 18	85 ± 10	94 ± 9	101 ± 5	8	17	19	11	10	5	0.01	0.04	0.9902
**UV 329**	82 ± 23	54 ± 10	101 ± 29	100 ± 11	101 ± 10	99 ± 8	75 ± 21	41 ± 7	85 ± 24	96 ± 11	103 ± 11	99 ± 8	8	26	23	11	10	8	0.03	0.10	0.9949
**UV 531**	115 ± 13	64 ± 9	83 ± 25	96 ± 17	94 ± 14	99 ± 12	132 ± 16	51 ± 7	78 ± 23	85 ± 15	82 ± 12	99 ± 12	12	15	26	18	15	12	0.03	0.08	0.9972

When internal standard calibration was initially applied for quantification in GC–MS using allyl-benzotriazole as surrogate standard, low recoveries were obtained for the benzotriazole UV stabilizers at medium and high fortification concentrations. Matrix-matched calibration was therefore employed in the GC–MS method for quantitation of benzotriazole UV stabilizers in method validation samples and real wastewater samples. Matrix effect was evaluated for the GC–MS method at the six different fortification levels. As shown in Fig. S2 (Supplementary material), ion suppression or enhancement was low (< 25%) for all studied benzotriazole UV stabilizers, except at the low spiking concentration of 0.02 ng mL^−1^ where ion enhancement was evident for most of the benzotriazole UV stabilizers.

### Application to real wastewater samples

The developed methods were successfully applied to determine target UV filters and benzotriazole UV stabilizers in a wastewater treatment plant in Lüneburg, Germany. Measured concentrations in influent and effluent are presented in Table 5. Only effluent was collected during the first sampling, while influent and effluent were collected in the second sampling**.** In the first sampling campaign, AVO and BP3 were measured in the effluent at 0.10 ng mL^−1^ and 0.12 ng mL^−1^, respectively while all other UV filters were determined below their limits of quantification. However, in the second sampling 4-MBC was measured up to 0.12 ng mL^−1^ in the effluent in addition to AVO and BP3 which were measured in the effluent of the first sampling. Again, the remaining UV filters were determined below their limits of quantification in the effluent of the second sampling. Meanwhile, all the UV filters were quantified in the influent (collected during the second sampling) at concentrations above their respective limit of quantification. Generally, the highest mean concentration was determined for OCR in influent (3.493 ng mL^−1^). High concentrations of OCR were also found both in influent by Balmer et al., 2005 (around 12 ng mL^−1^) and effluent wastewater by Langford et al., 2015 (near 7 ng mL^−1^). Other UV filters with high mean concentrations in influent were EHS (1.88 ng mL^−1^) and EHMC (0.620 ng mL^−1^). BP 3 was measured in influent (0.40 ng mL^−1^) at the same order of concentration levels quantified in Germany by Rodil et al., 2009 (0.234 ng mL^−1^) and Wick et al., 2010 (0.195 ng mL^−1^). However, the UV filters (especially OCR, EHMC and BP3) were measured in effluents at lower concentrations than the levels found in effluent by Moeder et al. (2010) in Germany.

**Table 5:** Measured concentrations (Mean ± SD) in influent and effluents from a WWTP in Lüneburg, Germany^a^

Compound	1st sampling	2nd sampling		Instrumental method of quantitation
	Effluent n = 4 (ng mL^−1^)	Influent n = 4 (ng mL^−1^)	Effluent n = 4 (ng mL^−1^)	
AVO	0.10 ± 0.01	0.26 ± 0.08	0.09 ± 0.01	LC–MS/MS
BP3	0.12 ± 0.02	0.40 ± 0.10	0.12 ± 0.01	LC–MS/MS
EHMC	< LOQ	0.62 ± 0.14	< LOQ	LC–MS/MS
EHS	< LOQ	1.88 ± 0.28	< LOQ	LC–MS/MS
4-MBC	< LOQ	0.16 ± 0.01	0.12 ± 0.04	LC–MS/MS
OCR	< LOQ	3.49 ± 1.19	< LOQ	LC–MS/MS
ODPABA	< LOQ	0.25 ± 0.04	< LOQ	LC–MS/MS
PABA	< LOQ	0.25 ± 0.07	< LOQ	LC–MS/MS
UV P	< LOQ	< LOQ	< LOQ	GC–MS
UV 234	< LOQ	< LOQ	< LOQ	GC–MS
UV 326	< LOQ	0.32 ± 0.06	< LOQ	GC–MS
UV 327	< LOQ	< LOQ	< LOQ	GC–MS
UV 328	0.06 ± 0.00	0.08 ± 0.01	0.06 ± 0.00	GC–MS
UV 329	< LOQ	0.11 ± 0.02	< LOQ	GC–MS
UV 531	< LOQ	0.44 ± 0.17	< LOQ	GC–MS

^a^ < LOQ- below method limit of quantification

ODPABA was found in influent at a mean concentration of 0.25 ng mL^−1^. Similar concentrations of ODPABA were quantified in influent by Tsui et al. (2014) in Hong Kong, but other researchers measured lower concentrations of ODPABA (Kameda et al., 2011; Magi et al., 2013; Rodil et al., 2012).

Benzotriazole UV stabilizers were also determined in effluent samples below the limit of quantification during the two samplings, except for UV 328. Same mean concentrations (0.06 ng mL^−1^) were observed in effluent for UV 328 in both first sampling and second sampling. Higher amount of UV 328 (0.08 ng mL^−1^) was, however, measured in influent. Except for UV P, UV 234 and UV 326, the target UV stabilizers were all quantified in the influent above their respective limit of quantification, with UV 531 having the highest mean concentration of 0.44 ng mL^−1^. Similar concentration to the level determined in this study were measured for UV 328 by Carpinteiro et al. (2012), but lower level was measured for this benzotriazole UV stabilizer in influent by Zhao et al. (2017) in China. However, these benzotriazole UV stabilizers were not detected in wastewater from some other studies (Wang et al., 2018). This work detected a rarely studied UV filter (PABA) in WWTP wastewater in influent and effluent. This compound (PABA) was also detected in drain water samples from a waste disposal site of the former ammunition plant in Stadtallendorf, Germany (Schmidt et al., 1997). Like most other UV filters, PABA has relative stability against biotic degradation and is, therefore, difficult to remove by conventional treatment methods (Díaz-Cruz & Barceló, 2009; Xue et al., 2015). In addition, only one study (Li et al., 2017) quantified 2-hydroxy-4-octyloxybenzophenone (UV 531) in wastewater matrix to date. Moreover, the occurrence of most of the target benzotriazole UV stabilizers (UV P, UV 234, UV 326, UV 327, UV 328, UV 329) in wastewater from a WWTP in Germany has been demonstrated in this work for the first time. This probably indicates the application of these compounds as UV stabilizers in textiles and technical products, such as plastics and paints, used in Germany. It should be noted that grab samples were collected for the methods development and the applicability of the methods. The results presented herein only demonstrate a glimpse of the occurrence of target UV absorbents in wastewater from Lüneburg WWTP. Further sampling campaigns involving the collection of 24 h composite samples during more extended sampling periods are recommended. Moreover, as most of the compounds in this study have a fairly high log Kow value, a significant portion can be adsorbed to the suspended solids. Therefore, analysis of particulates matter should also be included.

### Environmental risk assessment

**Fig. 5:**
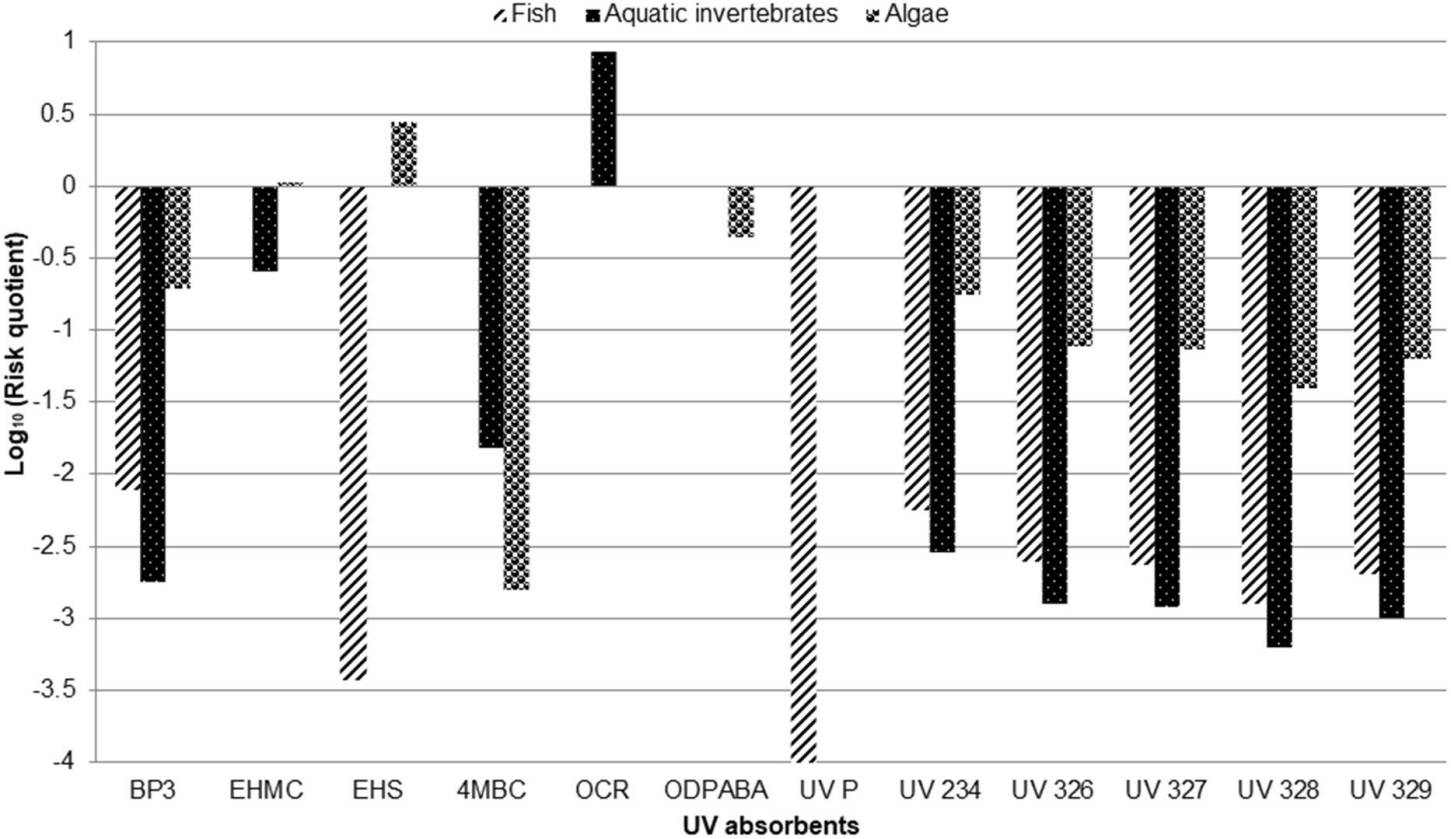
Risk quotients of UV filters and benzotriazole UV stabilizers, NB: Risk quotient values refer to effluent

The calculated risk quotient values are presented in Table S2, while Fig. 5 shows the logarithm (to base 10) of the risk quotient values. Among the UV filters, EHMC and EHS posed high risks to algae while only OCR presented high risk to aquatic invertebrates. BP3 and ODPABA presented a moderate risk to algae whereas EHMC posed moderate risk to aquatic invertebrates. Moreover, both BP3 and EHS posed a negligible risk to fish. Out of the benzotriazole UV stabilizers, only UV 234 posed a moderate risk to algae while others posed low risks to algae. All studied benzotriazole UV stabilizers presented negligible risks to aquatic invertebrates and fish. Though the risk quotients calculated for the lipophilic benzotriazole UV stabilizers in this study may be low (< 1 in all cases), the measured environmental concentrations could still pose a risk to aquatic organisms and species at higher trophic levels as a result of their potential bioaccumulation and biomagnification in the marine food web (Apel et al., 2018b).

## Conclusions

Analytical methods which allow the simultaneous extraction of fifteen multiclass UV filters and stabilizers (UV absorbents) from wastewater using an optimized single solid phase extraction protocol for both LC–MS/MS and GC–MS analyses were presented. The methods were successfully applied to determine target UV filters and benzotriazole UV stabilizers in wastewater from a WWTP in Lüneburg, Germany. All target UV absorbents were detected in influent and effluent wastewater. The highest mean concentration was determined for OCR in influent (3.49 ng mL^−1^). Detection of rarely investigated PABA and UV 531 (BP-12) in WWTP influent and effluent is reported. For the first time, the occurrence of most of the target benzotriazole UV stabilizers (UV P, UV 234, UV 326, UV 327, UV 328, UV 329) in wastewater from a German WWTP was demonstrated. The assessment of potential ecological risk shows that EHMC and EHS posed high risks to algae while only OCR presented a high risk to aquatic invertebrates. All benzotriazole UV stabilizers presented negligible risks to fish and aquatic invertebrates. This work provides new insight into the occurrence and potential risk of these UV filters and benzotriazole UV stabilizers in wastewater from WWTPs.

## Supplementary information

Below is the link to the electronic supplementary material.

Supplementary file1 (DOCX 488 KB)

## Data Availability

Data is provided within the manuscript or supplementary information files.
