# Supplementary material for: Determination and risk assessment of UV filters and benzotriazole UV stabilizers in wastewater from a wastewater treatment plant in Lüneburg, Germany
Source: Environ Monit Assess. 2024 Jul 11;196(8):725. doi: 10.1007/s10661-024-12853-2 (PMC11485089; doi:10.1007/s10661-024-12853-2)
Supplement: Supplementary file 1 — Supplementary file1 (DOCX 488 KB) [file 10661_2024_12853_MOESM1_ESM.docx]

**Supplementary Information**

**Determination and risk assessment of UV filters and benzotriazole UV stabilizers in wastewater from a wastewater treatment plant in Lüneburg, Germany**

Akinranti S. Ajibola^a,b*^, Marco Reich^a^, Klaus Kümmerer^a*^

^a^Institute of Sustainable Chemistry, Leuphana University Lüneburg, Universitätsallee 1, 21335 Lüneburg, Germany

^b^Analytical/Environmental Unit, Department of Chemistry, University of Ibadan, Ibadan, Nigeria

*Corresponding author: Akinranti S. Ajibola ([akinrantiaji@gmail.com; as.ajibola@mail.ui.edu.ng](mailto:akinrantiaji@gmail.com;%20as.ajibola@mail.ui.edu.ng); ORCID ID: [0000-0003-1682-7697](https://orcid.org/0000-0003-1682-7697))

Klaus Kümmerer (klaus.kuemmerer@leuphana.de, ORCID ID: 0000-0003-2027-6488)

**CONTENTS**

**Initial SPE procedure during optimization using ultrapure water**

**Table S1:** Structures and physicochemical properties of target UV filters and benzotriazole UV stabilizers

**Table S2:** Toxicity data and calculated risk quotient of target UV filters and benzotriazole UV stabilizers

**Table S3**: Efficiency of SPE cartridges tested during optimization with ultrapure water (LC-MS/MS analysis)

**Table S4** Efficiency of SPE cartridges tested during optimization with ultrapure water (GC-MS analysis

**Fig. S1**: Applicability of octocrylene-d_15_ (OCR-d_15_) as internal standard for matrix effect reduction in LC-MS/MS

**Fig. S2:**  Matrix effects in GC-MS method at different fortification levels

**References**

**Initial SPE procedure during optimization using ultrapure water**

Solid phase extraction sorbent was conditioned with 5 mL ethylacetate/dichloromethane (1:1), 5 mL methanol, 5 mL ultrapure water. 200 mL ultrapure water sample, spiked with a mixture of all analytes at initial concentration of 10 ng mL^-1^, was loaded on the cartridge. Cartridges were washed with 5 mL ultrapur**e** water, dried for 10 minutes under vacuum. Elution was carried out with 10 mL (2 mL x 5) ethylacetate/dichloromethane (1:1). The collected 10 mL of elution solvent was transferred into a centrifuge tube, vortex mixed and divided into 2 (5 mL each for LC-MS/MS analysis and GC-MS). The extract was evaporated to dryness under a gentle stream of nitrogen. Final extract was reconstituted with 1 mL acetonitrile/0.1%f formic acid in water (1:1) in the case of LC-MS/MS determinations and 1 mL of n-hexane for GC-MS analyses.

**Table S1:** Structures and physicochemical properties of target UV absorbents

| **Compound** | **CAS No** | **Structure** | **Chemical class** | **Molecular**  **Weight** | **Log K_ow_** | **UV absorbent type** |
| --- | --- | --- | --- | --- | --- | --- |
| OCR | 6197-30-4 | 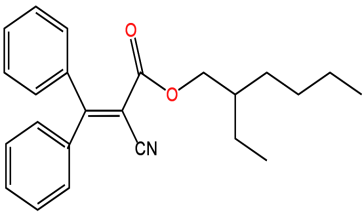 | Crylene derivative | 361.48 | 6.9^a^ | UV filter |
| EHMC | 5466-77-3 | 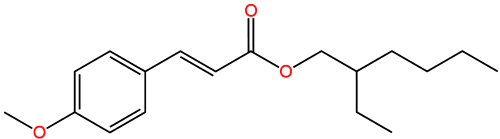 | Cinnamates derivative | 290.40 | 5.80^a^ | UV filter |
| BP3 | 131-57-7 | 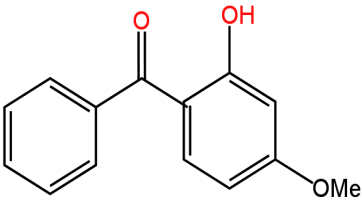 | Benzophenone derivative | 228.24 | 3.5^a^ | UV filter |
| 4-MBC | 36861-47-9 | 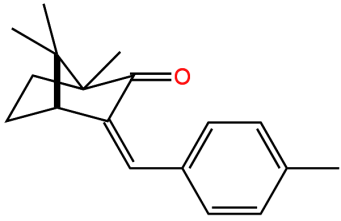 | Camphor derivative | 254.37 | 5.9^a^ | UV filter |
| EHS | 118-60-5 | 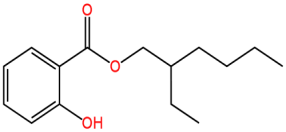 | Salicylate derivative | 250.33 | 6.0^a^ | UV filter |
| PABA | 150-13-0 | 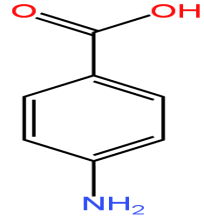 | p-amino benzoic acid derivative | 137.14 | 0.83^c^ | UV filter |
| ODPABA | 21245-02-03  58817-05-3 | - 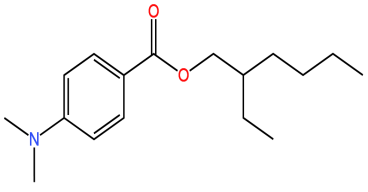 | p-amino benzoic acid derivative | 277.40 | 5.8^a^ | UV filter |
| AVO | 70356-09-1 | 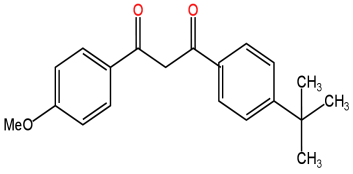 | Dibenzoyl methane derivative | 310.39 | 4.3^a^ | UV filter |
| UV 327 | 3864-99-1 | 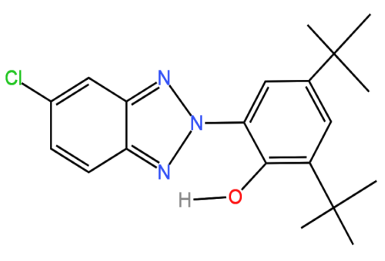 | Benzotriazole derivative | 357.88 | 6.9^a^ | UV stabilizer |
| UV 328 | 25973-55-1 | 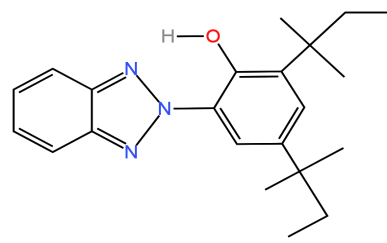 | Benzotriazole derivative | 351.49 | 7.3^a^ | UV stabilizer |
| UV 326 | 3896-11-5 | 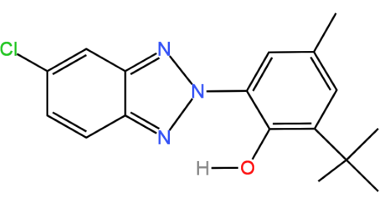 | Benzotriazole derivative | 315.80 | 5.6^a^ | UV stabilizer |
| UV 531 | 1843-05-6 | 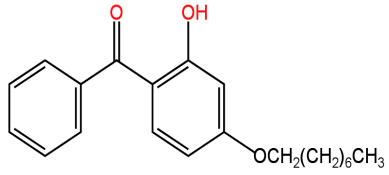 | Benzophenone derivative | 326.43 | 6.42^b^ | UV stabilizer/  plasticizer |
| UV 329 | 3147-75-9 | 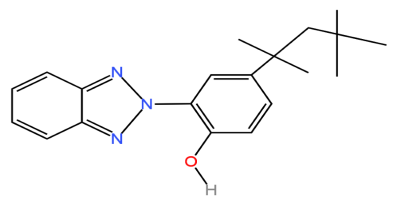 | Benzotriazole derivative | 323.43 | 6.2^a^ | UV stabilizer |
| UV 234 | 70321-86-7 | 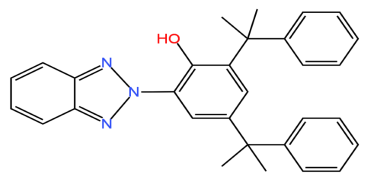 | Benzotriazole derivative | 447.57 | 7.7^a^ | UV stabilizer |
| UV P  UV P | 2440-22-4 | 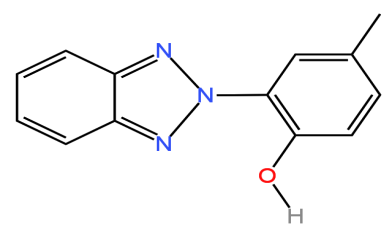 | Benzotriazole derivative | 225.25 | 3.0^a^ | UV stabilizer |
| OCR–d_15_ | - | 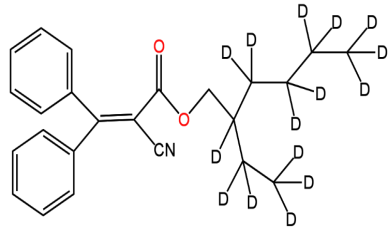 | Crylene derivative | 376.57 | - |  |
| Allyl-benzotriazole | 2170-39-0 | 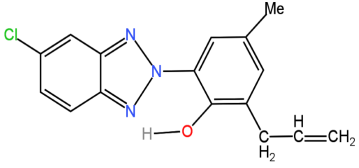 | Benzotriazole derivative | 265.31 | 1.98^e^ |  |

a= Apel et al. 2018

b =Peng et al. 2017

c= Hansch et al. 1995

d= <https://chemicalize.com/#/calculation>

e = [www.chemspider.com](http://www.chemspider.com)

**Table S2:** Toxicity data and calculated risk quotient of target UV absorbents

| Compound | **^a^Toxicity value (µg/L)** | | | **Assessment factor** | **PNEC**  **(ng/L)** | | | **MEC**  **(ng/L)** | **^b^RQ** | | |
| --- | --- | --- | --- | --- | --- | --- | --- | --- | --- | --- | --- |
|  | Fish | Aquatic invertebrates | Algae |  | Fish | Aquatic invertebrates | Algae |  | Fish | Aquatic invertebrates | Algae |
| BP3 | 749 | 3280 | 30 | 50 | 14980 | 65600 | 600 | 117 | 0.008 | 0.002 | 0.195 |
| EHMC | - | 62.5 | 15 | 100 | 0 | 625 | 150 | 159 | nc | 0.254 | **1.060** |
| EHS | 82000 | - | 11 | 100 | 820000 | nc | 110 | 307 | 0.0004 | nc | **2.791** |
| 4-MBC | - | 800 | 7660 | 100 | nc | 8000 | 76600 | 122 | nc | 0.015 | 0.002 |
| OCR | - | 3.18 | - | 100 | nc | 31.8 | nc | 277 | nc | **8.711** | nc |
| ODPABA | - | - | 25 | 100 | nc | nc | 250 | 111 | nc | nc | 0.444 |
| UV P | 100000 | - | - | 100 | 1000000 | nc | nc | 103 | 0.00010 | nc | nc |
| UV 234 | 5000 | 10000 | 160 | 100 | 50000 | 100000 | 1600 | 285 | 0.006 | 0.003 | 0.178 |
| UV 326 | 5000 | 10000 | 160 | 100 | 50000 | 100000 | 1600 | 125 | 0.003 | 0.001 | 0.078 |
| UV 327 | 5000 | 10000 | 160 | 100 | 50000 | 100000 | 1600 | 119 | 0.0024 | 0.0012 | 0.074 |
| UV 328 | 5000 | 10000 | 160 | 100 | 50000 | 100000 | 1600 | 63 | 0.001 | 0.001 | 0.039 |
| UV 329 | 5000 | 10000 | 160 | 100 | 50000 | 100000 | 1600 | 102 | 0.002 | 0.001 | 0.064 |

^a^Data obtained from Allinson et al. (2018); ^b^Risk quotient values refer to effluent; nc- not calculated due to non-availability of toxicity data

**Table S3**: Efficiency (absolute recovery) of SPE cartridges tested during optimization with ultrapure water (LC-MS/MS analysis), final concentration in extract = 1000 ng mL^-1^

|  | **Chromabond HLB Cartridge** | | **Strata X-CW Cartridge** | | **Chromabond HLB Cartridge** | **Strata X-CW Cartridge** |
| --- | --- | --- | --- | --- | --- | --- |
| **Compound** | SPE Recovery | Overall recovery | SPE Recovery | Overall recovery | Matrix effect | Matrix effect |
| **AVO** | 47±10 | 38±4 | 42±9 | 38±6 | -18 | -7 |
| **BP3** | 100±23 | 79±3 | 74±1 | 65±3 | -19 | -13 |
| **EHMC** | 58±16 | 50 ±1 | 51±6 | 47±6 | -11 | -6 |
| **EHS** | 50±6 | 57±6 | 36±6 | 33±4 | -9 | -7 |
| **4-MBC** | 98±25 | 80±4 | 69±6 | 62±6 | -16 | -10 |
| **OCR** | 29±10 | 27±2 | 29±5 | 27±4 | -1 | -5 |
| **ODPABA** | 70±5 | 55±1 | 54±5 | 50±6 | -21 | -8 |
| **PABA** | 32±11 | 30±11 | 81±8 | 53±4 | -28 | -34 |

**Table S4**: Efficiency (absolute recovery) of SPE cartridges tested during optimization with ultrapure water (GC-MS analysis);

|  | **Chromabond HLB Cartridge** | | **Strata X-CW Cartridge** | | **Chromabond HLB Cartridge** | **Strata X-CW Cartridge** |
| --- | --- | --- | --- | --- | --- | --- |
| **Compound** | SPE Recovery | Overall recovery | SPE Recovery | Overall recovery | Matrix effect | Matrix effect |
| **Allyl-benzotriazole** | 63±14 | 58±16 | 75±14 | 103±16 | -9 | +37 |
| **UV P** | 94±26 | 82±25 | 87±17 | 106±21 | -13 | +22 |
| **UV 234** | 0.9±0.5 | 1.1±0.7 | 0.8±0.1 | 1.5±0.1 | +19 | +77 |
| **UV 326** | 5±1 | 4±1 | 5±1 | 7.5±0.3 | -16 | +37 |
| **UV 327** | 2±1 | 1.3±0.6 | 1.5±0.2 | 1.9±0.3 | -23 | +28 |
| **UV328** | 17±3 | 14±3 | 2±1 | 3±1 | -19 | +31 |
| **UV329** | 10±2 | 8±2 | 13±1 | 17±1 | -15 | +36 |
| **UV 531** | 6±2 | 7±3 | 6±2 | 11±1 | +14 | +91 |

10 ng mL^-1^ fortification level (final extract concentration = 1000 ng mL^-1^), 200 mL ultrapure water

**Fig. S1**: Applicability of OCR-d_15_ as internal standard for matrix effect reduction in LC-MS/MS

**Fig. S2:**  Matrix effects in GC-MS method at different fortification levels

**References**

Allinson, M., Kameda, Y., Kimura, K., & Allinson, G. (2018). Occurrence and assessment of the risk of ultraviolet filters and light stabilizers in Victorian estuaries. *Environmental Science & Pollution Research*, *25 (12*), 12022–12033. <https://doi.org/10.1007/s11356-018-1386-7>.

Apel, C., Joerss, H., & Ebinghaus, R. (2018). Environmental occurrence and hazard of organic UV stabilizers and UV filters in the sediment of European North and Baltic Seas. *Chemosphere,* *212*, 254–261. <https://doi:10.1016/j.chemosphere.2018.08.105>

Peng, X., Xiong, S., Ou, W., Wang, Z, Tan, J., Jin, J., Tang, C., Liu, J., & Fan, Y. (2017). Persistence, temporal and spatial profiles of ultraviolet absorbents and phenolic personal care products in riverine and estuarine sediment of the Pearl River catchment, China. *Journal of Hazardous Materials*, *323*, 139-146. <https://doi.org/10.1016/j.jhazmat.2016.05.020>.

Hansch, C., Leo, A., & Hoekman, D. (1995). Exploring QSAR- Hydrophobic, Electronic and Steric Constants. ACS Professional Reference Book. American Chemical Society, Washington (DC, USA)
